# A study on the monitoring of heatwaves and bivariate frequency analysis based on mortality risk assessment in Wuhan, China

**DOI:** 10.3389/fpubh.2024.1409563

**Published:** 2024-06-19

**Authors:** Si Chen, Junrui Zhao, Haonan Dou, Zhaoqian Yang, Fei Li, Jihye Byun, Seong Wook Kim

**Affiliations:** ^1^School of Resources and Environmental Science, Hubei University, Wuhan, China; ^2^Hubei Key Laboratory of Regional Development and Environmental Response, Hubei University, Wuhan, China; ^3^School of Information and Safety Engineering, Zhongnan University of Economics and Law, Wuhan, China; ^4^College of Earth and Environmental Sciences, Lanzhou University, Lanzhou, China; ^5^Department of Transportation Engineering, University of Seoul, Seoul, Republic of Korea; ^6^Department of Applied Mathematics, Hanyang University, Ansan, Republic of Korea

**Keywords:** heatwave risk, copula function, global climate models, co-occurrence return periods, Wuhan city

## Abstract

The increasingly frequent occurrence of urban heatwaves has become a significant threat to human health. To quantitatively analyze changes in heatwave characteristics and to investigate the return periods of future heatwaves in Wuhan City, China, this study extracted 9 heatwave definitions and divided them into 3 mortality risk levels to identify and analyze historical observations and future projections of heatwaves. The copula functions were employed to derive the joint distribution of heatwave severity and duration and to analyze the co-occurrence return periods. The results demonstrate the following. (1) As the concentration of greenhouse gas emissions increases, the severity of heatwaves intensifies, and the occurrence of heatwaves increases significantly; moreover, a longer duration of heatwaves correlated with higher risk levels in each emission scenario. (2) Increasing concentrations of greenhouse gas emissions result in significantly shorter heatwave co-occurrence return periods at each level of risk. (3) In the 3 risk levels under each emission scenario, the co-occurrence return periods for heatwaves become longer as heatwave severity intensifies and duration increases. Under the influence of climate change, regional-specific early warning systems for heatwaves are necessary and crucial for policymakers to reduce heat-related mortality risks in the population, especially among vulnerable groups.

## 1 Introduction

The sixth assessment report by the Intergovernmental Panel on Climate Change stated that the use of fossil fuels and unsustainable land use have contributed to global warming; the global average surface temperature between 2011 and 2020 has been reported to be about 1.1°C hotter than the pre-industrial level, and the frequency of high heat episodes per 50-year period increased by 4.8 times (1). Expanding urban land use will encroach on ecological space such as grasslands, cropland and unutilized land, and this unsustainable land-use competition will exacerbate the occurrence of heatwave events (2). Consistent with anthropogenically driven climate warming, the frequency and severity of extreme temperatures and heatwaves worldwide are expected to increase in the coming decades, posing pervasive threats to both human systems and ecosystems and generally having adverse effects on human physical and mental health, livelihoods, infrastructure, and a range of other global aspects (3). Almost half the global population is likely to be exposed to life-threatening temperature extremes annually by 2,100 (4). It was estimated that, in 43 countries globally from 2000 to 2019, 9.43% of deaths per year were attributable to temperatures that were either too low or too high, and excess deaths due to high temperatures accounted for 0.91% of the total (5). One of the most typical heatwaves of the 21st century was the 2003 heatwave in Central and Western Europe, which caused more than 70,000 deaths and illustrated the importance of heatwave disasters (6). Increase in the frequency and intensity of heatwaves also can lead to a rapid escalation in energy consumption. Extreme weather events such as droughts and heat waves exacerbate water scarcity by reducing water availability, deteriorating water quality and increasing sectoral water use (7). Additionally, the power sector is particularly vulnerable to climate change, and heatwaves increase the cooling demand, which affects residential, commercial, and industrial electricity demand (8). A case study in Shanghai found that a 1°C increase in global mean temperature could lead to a 36.1% surge in annual peak electricity use (9). Luo et al. (10) found that slowdown in the movement of heat waves in recent decades could increase the risk of reduced productivity of local ecosystems and increase consumption and capacity needs in the energy sector. Similarly, human health is affected by severe heatwaves. In China, a study found that the number of deaths caused by heatwaves has increased rapidly since 1979, and although factors such as population growth, an aging population, and rising baseline mortality rates have objectively contributed to the number of deaths caused by heatwaves, the rapid increase in the frequency of heatwaves has been the most significant contributing factor (11).

Currently, there is no consistent definition of heatwave, which is generally defined as consecutive days above a certain temperature threshold, but studies on heatwaves are mostly based on temperature indicators (or thresholds) and duration. China’s meteorological department defines a heatwave as 3 or more consecutive days with a daily maximum temperature exceeding 35°C (12), and the World Meteorological Organization defines a heatwave as a daily maximum temperature above 32°C on 3 or more consecutive days (13). However, the vulnerability of populations in different regions exposed to heatwaves varies according to long-term adaptation to the surrounding climate (14). Therefore, a consistent and standard definition of heatwaves is not well applied in China, which has a diverse range of climate types. Several studies have defined heatwaves using temperature percentiles that consider the local climatic characteristics as the thresholds. A heatwave health risk study in Australia defined heatwaves as 2 or more consecutive days on which the average temperature exceeds a particular percentile of the warm season average (15). A study on heatwaves in 31 provincial capital cities in China proposed a definition of a daily maximum temperature ≥ 92.5 percentile for a duration ≥3 days (16). Yin et al. (17) collected the daily mean temperatures of 272 major cities in China from 2013 to 2015 and then combined the 90th, 92.5th, 95th, and 97.5th percentiles and durations of 2, 3, and 4 days to construct 12 definitions of heatwaves; they then investigated the characterization of all 12 heatwave types and calculated the risk of mortality associated with each type for different subgroups of the population. In addition, heat wave events have spatial continuity, with heat wave propagation distances, movement speeds and directions changing over time (18). Recognizing the movement patterns and propagation cycles of heat waves can provide potential precursor signals, and understanding their co-evolution in both temporal and spatial dimensions is important for understanding heat wave prediction, mitigation and adaptation (10).

Researchers have described the conditions of heatwaves based on the characteristics of severity and duration, and those 2 characteristics play an important role in the heatwave frequency analyses used to develop comprehensive predictions. Given that a heatwave is a multivariate phenomenon, the recurrence interval of heatwave severity and duration could be quite different, even if both characteristics were obtained from a single event (19, 20). Therefore, several studies have suggested using multivariate methods, especially copula functions with joint frequency analyses, to assess the severity and duration of heatwaves. In a study of heatwaves in the Yangtze River Delta, Xu et al. introduced a copula function to investigate the heatwave characteristics of 2 probabilistic models for a specific period (21). Mazdiyasni et al. (22) used a copula function to compare the intensity-epoch-frequency curves of heatwaves in 6 cities in the United States and to find heatwave hazards and their joint recurrence periods in different regions. Copula functions have been used to develop bivariate joint probability density functions based on the marginal probability distribution of each variable, which allow the heatwave characteristics and changes in future periods to be detected by combining them with climate change scenarios. Such functions can provide optimal management conditions for each region. Heatwaves pose a threat to urban economies and the health of residents, and the construction of a heatwave early-warning system can mitigate the health risks of populations exposed to heatwaves (23).

Dong et al. (24) proposed a heatwave health risk framework based on the Heat Climate Index and assessed its applicability to 177 neighborhoods in Wuhan City, China. Similarly, Zhang et al. (25) combined high-temperature heatwave data and health risk data to develop an early warning model. The investigation of changes in heatwaves during a future period and the stratification of heatwave risks can facilitate the development of heatwave early warning systems. In China, the Meteorological Bureau issues heat warning signals by classifying heatwave grades based on the heatwave index (26), but it neglects the risk level in terms of the relationship between a heatwave and the mortality exposure response of the population. Thus, the analysis and prediction of heatwaves under future climate patterns provide a scientific foundation for the formulation of a rational national development strategy. A series of national and international articles has examined assessments of future temperature changes from new typical concentration pathway scenarios under multiple future climate models. Yun et al. (27) evaluated the simulated outcomes of 27 climate models from the fifth Coupled Model Intercomparison Project (CMIP5) and considered the extent of warming in Asia under multiple emission pathways. In contrast, Brown et al. (28) developed a new statistical model to predict changes in heatwave intensity over time for 20 cities around the world using the RCP8.5 emissions scenario for 28 CMIP5 climate models. Few current studies have considered the frequency of heatwaves under future climate models, and they all show that heatwave events will occur more frequently in the future, but they have not examined heatwave frequency in terms of heatwave features (29–31).

Therefore, based on daily maximum temperature data for Wuhan City from 1951 to 2017 and daily maximum temperature prediction data from 4 CMIP5 global climate models under 2 emission scenarios (RCP4.5 and RCP8.5) from 2031 to 2099, this study (1) constructs heatwave definitions for Wuhan by exploring the relationship between heatwaves and the mortality-exposure response of residents and combines those definitions to stratify the heatwave risk and to identify heatwave events with different risks; (2) fits the marginal distributions of historical heatwave characteristics in Wuhan and introduces a copula function to analyze the bivariate joint probability and co-occurrence return period; (3) predicts future heatwave co-occurrence periods in Wuhan, compares those predictions with historical heatwave events, analyzes changes in heatwave event characteristics, and provides a scientific basis for a response to climate change and risk management in Wuhan.

## 2 Study area and data sources

### 2.1 Study area

**Figure 1:**
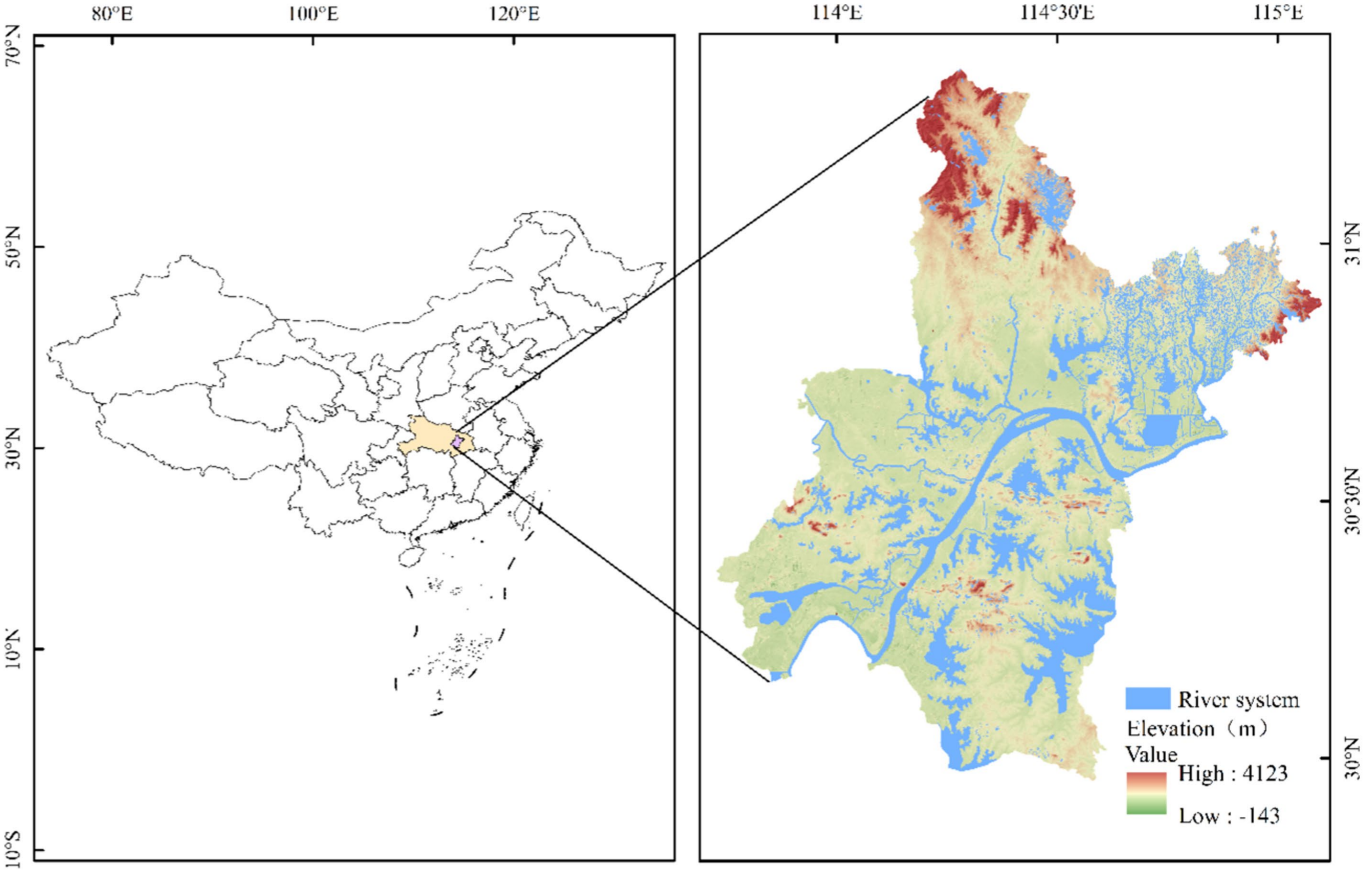
Geographical location of Wuhan.

Wuhan is the capital of Hubei Province (29°58′N to 31°22′N, 113°41′E to 115°05′E) and has a total area of 8569.15 km^2^ (Figure 1). Wuhan has a subtropical humid monsoon climate. Under the influence of tropical cyclones in the western part of the North Pacific Ocean, high temperatures are particularly likely to occur in the middle and lower reaches of the Yangtze River in east-central China (32). As the only supercity in that central region, Wuhan is a typical furnace city. In 2014, the maximum temperature in Wuhan reached 39.7°C. At the end of 2022, Wuhan had a resident population of 13.739 million and a gross domestic product of 1.89 trillion yuan. Because high temperatures cause death and economic losses (33), the need to analyze their characteristics in Wuhan and to develop plans to reduce their effects is urgent.

### 2.2 Data sources

This study uses daily maximum temperature data recorded at the Wuhan National Basic Meteorological Station (57494) from 1951–2017. These historical observational data were sourced from the National Meteorological Data Center of the China Meteorological Network.^1^ The future data for this study are the daily maximum near-surface air temperatures from 2007–2017 and 2031–2099 projected under 2 representative concentration pathways, RCP4.5 (medium emission scenario) and RCP8.5 (high emission scenario), for 4 CMIP5 climate models.^2^ Table 1 provides basic information about the 4 global climate models. The “r1i1p1” ensemble was selected for the global climate models used in this study, and all models were re-gridded to 0.5° × 0.5° resolution.

**Table 1:** Details of the CMIP5 global climate models.

ID	Model	Nation	Resolution
			(Longitude (°) × Latitude (°))
1	GFDL-ESM2M	USA	144 × 90
2	HadGEM2-ES	UK	145 × 192
3	IPSL-CM5A-LR	France	96 × 96
4	MIROC5	Japan	256 × 128

## 3 Methods

### 3.1 Definition-based heatwave stratification methods

Previous research combined temperature indicators, temperature thresholds, and durations to develop 45 definitions of heatwaves and to evaluate the effects of heatwave events on the mortality of Wuhan residents (34). In this study, 9 heatwave definitions were developed by combining 3 types of temperature thresholds and 3 durations, and then those definitions were divided into categories to represent low-, medium-, and high-risk events.

### 3.2 Joint probability analysis based on copulas

In this study, 6 commonly used distribution functions were selected to fit the marginal distributions of heatwave characteristics: the lognormal (Logn), exponential (Exp), gamma, generalized extreme value (GEV), Weibull (Wbl), and generalized pareto (GP) distributions. These 6 distribution functions were used to fit the marginal distributions of heatwave duration. The marginal distributions of heatwave severity were fitted using the first 5 distribution functions.

Copula functions are multivariate joint distribution functions that concatenate multiple univariate marginal distributions. Copulas are mainly based on the bivariate joint distribution, as shown in Eq. (1):

(1) $$F_{XY}\left(x,y\right)=C\left[F_{X}\left(x\right),F_{Y}\left(y\right)\right]$$

where *X* and *Y* are continuous variables, and C is the only copula function that fits the above equation. The variables of the copula function are the marginal distribution function of *X*, *Y*.

The copula functions used in this paper are the *t* copula, gaussian copula, Clayton copula, Gumbel copula, and Frank copula. The *t* copula has 2 parameters, and the rest of the copulas each have only 1 parameter. Both the optimal copula function and the optimal marginal distribution function were selected using the Multivariate Copula Analysis Toolbox.

### 3.3 Co-occurrence return periods

Return periods can provide effective support for decision-making about heatwave prevention and management. They are classified as joint and co-occurrence return periods, and this study uses the latter. The heatwave co-occurrence return periods require that the heatwave severity and duration simultaneously exceed specific values, and their calculation is based on 2 univariate marginal distributions and a bivariate joint distribution (35) as shown in Eq. (2):

(2) $$\begin{array}{c} T=\frac{N}{nP\left(X\geq x\cap Y\geq y\right)}\\ =\frac{N}{n\left\{1-F_{X}\left(x\right)-F_{Y}\left(y\right)+C\left[F_{X}\left(x\right),F_{Y}\left(y\right)\right]\right\}} \end{array}$$

where *N* is the length of the study time, and *n* is the number of heatwave events during the study time.

### 3.4 Global climate model assessment

Taylor diagrams assess the simulation capability of climate models (36) using the root mean square error (RMSE), correlation coefficient, and variance ratio (standard deviation), which are shown in Eqs. (3–6), respectively. Based on those indicators, a Taylor diagram can illustrate the matching ability of climate models with observations in terms of correlation, RMSE, and standard deviation, which intuitively reflects the matching performance between the simulated and observed data. Therefore, a Taylor diagram can comprehensively reflect the advantages and disadvantages of the simulation results of each model. In this paper, 4 global CMIP5 climate models and 2 representative concentration paths, RCP4.5 and RCP8.5, were selected to simulate the climate of Wuhan for assessments by Taylor diagram analyses.

(3) $$RMSE=\sqrt{\frac{1}{N}\overset{N}{\underset{n=1}{\sum }}{\left(o_{n}-m_{n}\right)}^{2}}$$

(4) $$R=\frac{1}{N}\overset{N}{\underset{n=1}{\sum }}\frac{\left(o-\bar{o}\right)\left(m-\bar{m}\right)}{\sigma_{o}\sigma_{m}}$$

(5) $$\sigma =\sqrt{\frac{1}{N}\overset{N}{\underset{n=1}{\sum }}{\left(x_{n}-\bar{x}\right)}^{2}}$$

(6) $$\sigma_{f}=\frac{\sigma_{m}}{\sigma_{o}}$$

where *o* and m are the observed and model-simulated data, respectively; $\bar{o}$ , $\bar{m}$ are the mean values of the observed and simulated data, and *σ_o_* and *σ_m_* are the standard deviations of the observed and simulated data. As *R* and *σ_f_* become closer to 1 and the RMSE becomes closer to 0, the model simulation is understood to improve.

It is difficult to discern the match between simulation results and observations when the simulation results of different climate models are close to one another in the Taylor diagram. Thus, to more objectively assess the quality of the temperature simulation from each climate model, the comprehensive skill score *S* is introduced (36) as shown in Eq. (7):

(7) $$S=\frac{4{\left(1+R\right)}^{4}}{{\left(\frac{\sigma_{m}}{\sigma_{o}}+\frac{\sigma_{o}}{\sigma_{m}}\right)}^{2}{\left(1+R_{0}\right)}^{4}}$$

where *R* is the correlation coefficient between model simulation values and observations, *σ_m_* is the standard deviation of the model simulation results, *σ_o_* is the standard deviation of the observations, and *R_0_* is the maximum value of the correlation coefficient between the model simulation results and observations.

## 4 Results

### 4.1 Definition and risk classification of heatwaves in Wuhan

**Figure 2:**
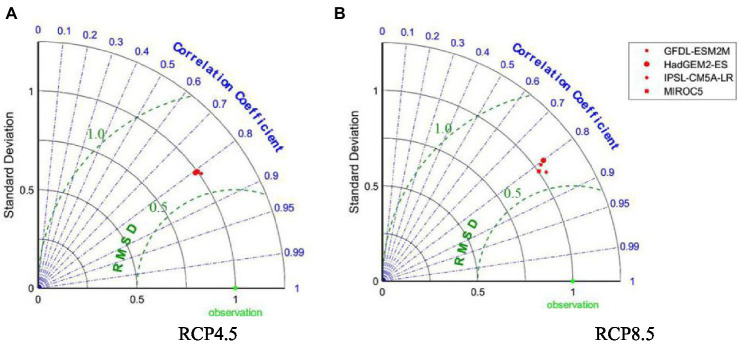
Taylor diagrams of 4 CMIP5 GCMs relative to observations under different emission scenarios in Wuhan, 2007–2017 **(A)** RCP4.5; **(B)** RCP8.5.

CMIP5 is a multi-model simulation of historical and future climate under different greenhouse gas emission scenarios (37). It provides a better understanding of present and projected future climate change. Despite moderate biases in day time temperatures, the CMIP5 model performs better in terms of frequency for the actual simulated observed heat waves (38). To assess the consistency between the 4 global climate models and the temperature in Wuhan under the 2 representative concentration pathways, the model projection data and observation data for the overlapping period between the 2 datasets (2007–2017) were compared, as shown in the Taylor diagrams in Figure 2. The correlation coefficients between the simulated results and the observed data under both the RCP4.5 and RCP8.5 emission scenarios were concentrated around 0.8. The ratio of the standard deviation of the simulated results to the observations under the RCP4.5 emission scenario was concentrated around 1, whereas that under the RCP8.5 emission scenario ranged from 1 to 1.06. The RMSE of the simulation results was centered at 0.62 under the RCP4.5 emission scenario and in the range of 0.58–0.63 under the RCP8.5 emission scenario.

In general, the simulation results of the 4 global climate models under the RCP4.5 emission scenario were clustered, which made it difficult to discern the applicability of each model. Under the RCP8.5 emission scenario, the IPSL-CM5A-LR and MIROC5 models were better than the GFDL-ESM2M and HadGEM2-ES models in all 3 metrics, but neither could be selected as optimal. Thus, the S-skill score was used to quantify the simulation quality of the 4 global climate models.

Table 2 demonstrates that under the RCP4.5 and RCP8.5 emission scenarios, the IPSL-CM5A-LR and MIROC5 models, respectively, best matched the Wuhan temperature observations. Under the RCP4.5 emission scenario, the 4 models matched Wuhan’s air temperature with little discrepancy. Under the RCP8.5 emission scenario, in contrast, the MIROC5 model had the best performance, and the HadGEM2-ES model had the worst performance. Therefore, the IPSL-CM5A-LR model and MIROC5 model were selected for further analysis under the RCP4.5 and RCP8.5 emission scenarios, respectively.

**Table 2:** Skill scores and rankings of the 4 CMIP5 GCMs in Wuhan under different emission scenarios, 2007–2017.

Model	RCP4.5 (S/ranking)	RCP8.5 (S/ranking)
GFDL-ESM2M	0.98/4	0.94/3
HadGEM2-ES	0.97/3	0.93/4
IPSL-CM5A-LR	0.99/1	0.97/2
MIROC5	0.98/2	0.99/1

To comprehensively demonstrate the association between mortality and heat waves, Zhang et al. (34) combined 5 temperature thresholds and 3 durations of the daily maximum temperature, minimum temperature, and mean temperature to develop 45 heatwave definitions for the selection of best definitions to capture the effects on non-accidental mortality in Wuhan. They found the intensity thresholds of the 95th percentile, 97.5th percentile, and 99th percentile of the daily maximum temperature, together with the duration ≥2 days, duration ≥3 days, and duration ≥4 days had good predictive ability in assessing the total mortality effects of heatwaves among Wuhan residents. Based on the previous study, we selected the daily maximum temperatures recorded by Wuhan meteorological stations (57494) from 1951–2017 and combined the corresponding 95th percentile, 97.5th percentile, and 99th percentiles with durations of ≥2 days, 3 days, and 4 days to develop 9 heatwave definitions, as shown in Table 3. These criteria were used to detect historical and future heatwave events in this study.

**Table 3:** The 9 heatwave definitions in Wuhan, 1951–2017.

Heatwave definition	Temperature indicator	Relative temperature threshold	Duration
HW1	Daily maximum temperature	95th percentile (35.2°C)	≥2d
HW2			≥3d
HW3			≥4d
HW4		97.5th percentile (36.2°C)	≥2d
HW5			≥3d
HW6			≥4d
HW7		99th percentile (37.1°C)	≥2d
HW8			≥3d
HW9			≥4d

**Figure 3:**
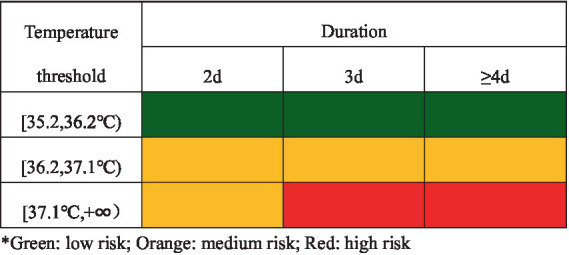
Heatwave mortality risk classification system.

Based on the 9 heatwave definitions extracted from the relationship between heatwave events and resident exposure mortality response in Wuhan, this paper proposes the heatwave mortality risk classification system shown in Figure 3. Low-risk heatwaves are events with a daily maximum temperature between 35.2 and 36.2°C for 2 days or more, as indicated in green. Medium-risk heatwaves are events with daily maximum temperatures between 36.2 and 37.1°C for 2 days or more or daily maximum temperatures of 37.1°C and higher for 2 days, as indicated in orange. High-risk heatwaves are events with daily maximum temperatures of 37.1°C and higher for 3 days or more, as indicated in red.

### 4.2 Characterization of historical heatwaves in Wuhan based on copula functions

**Figure 4:**
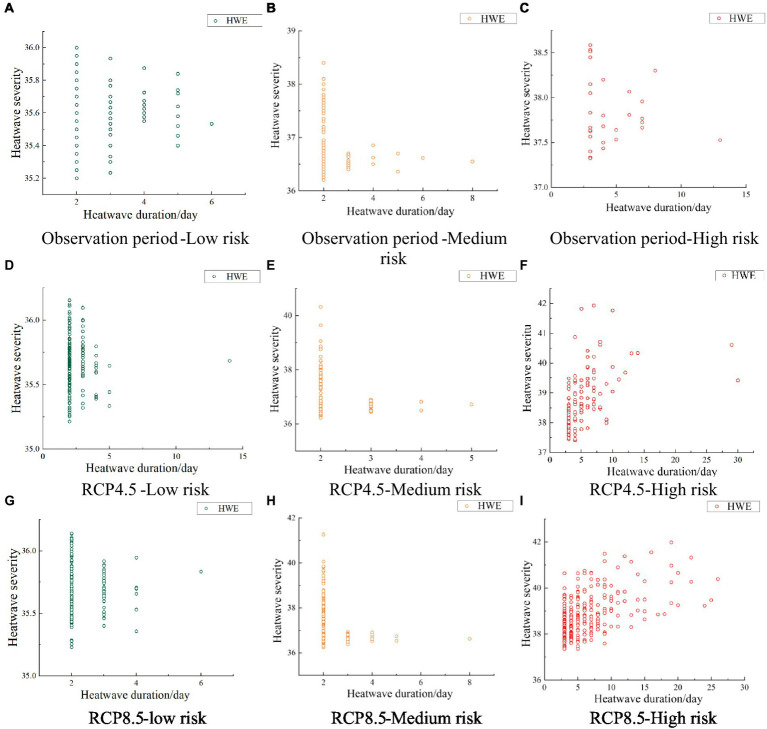
Scatterplots of heatwave severity and duration identified using different risk levels and RCP scenarios **(A, D, G)** low risk; **(B, E, H)** medium risk; **(C, F, I)** high risk.

Based on those tiered risk criteria, the 2 characteristic variables of heatwave severity and duration were used to classify observed (1951–2017) and predicted (2031–2099) heatwaves as low, medium, and high risk, as shown in Figure 4. In the different scenarios, the number of heatwave events increased significantly as the concentration of greenhouse gas emissions increased. The incidence of high-risk heatwaves increased exponentially, whereas the increase in the numbers of low-risk and medium-risk heatwave events was not obvious. Most of the scenarios showed that risk increases with heatwave duration. The number of heatwave occurrences differed among scenarios, with a decreasing trend in the observation period and RCP4.5 emission scenarios and an increasing trend in the RCP8.5 emission scenario.

In general, when the heatwave duration was shorter, the frequency increased. During the observation period, the maximum heatwave severity when the heatwave duration was 2d was 36°C and 38.4°C for the low- and medium-risk categories, respectively, and when the heatwave duration was 3d, the maximum heatwave severity was 38.586°C for the high-risk category. In the RCP4.5 scenario, the maximum heatwave severity when the heatwave lasted 2d was 36.155°C and 40.315°C for the low-risk and medium-risk categories, respectively, and the maximum heatwave severity for the high-risk category was 41.93°C in a heatwave predicted to last 7d. In the RCP8.5 scenario, heatwaves that lasted for 2d had a maximum severity of 36.14°C and 41.26°C for the low-risk and medium-risk categories, respectively, and the maximum heatwave severity for the high-risk category was 41.97°C in a heatwave predicted to last 19d. In other words, except for the RCP4.5 and RCP8.5 high-risk category, heatwave severity was highest when the heatwave lasted for the shortest time. The severity of the most intense heatwaves also tended to increase within each risk level in the different scenarios, indicating that an increase in the concentration of greenhouse gas emissions would lead to more intense heatwave events.

The severity of the heatwaves in the historical period was fitted using 5 marginal distribution functions (Logn, Exp, Gamma, GEV, and Wbl). In fitting the duration, the GP distribution was added. The results of the fitting are shown in Table 4. The table indicates that the optimal marginal distributions for the severity (S) and duration (D) of heatwaves during the historical observation period were GEV and GP, respectively. These are both 3-parameter distributions and were estimated using the maximum likelihood estimation method.

**Table 4:** Marginal distributions of heatwave characteristics under different risk levels.

Risk level	Characteristic	Marginal distribution	Parameters*
Low risk	S	GEV	[*k*,*σ*,*μ*] = [−0.29,0.17,35.53]
	D	GP	[*k*,*σ*,*μ*] = [−0.64,3.86,0]
Medium risk	S	GEV	[*k*,*σ*,*μ*] = [0.27,0.25,36.56]
	D	GP	[*k*,*σ*,*μ*] = [−0.34,2.93,0]
High risk	S	GEV	[*k*,*σ*,*μ*] = [0.07,0.28,37.66]
	D	GP	[*k*,*σ*,*μ*] = [−0.43,6.27,0]

*k: shape parameter, *σ*: scale parameter, *μ*: location parameter.

**Figure 5:**
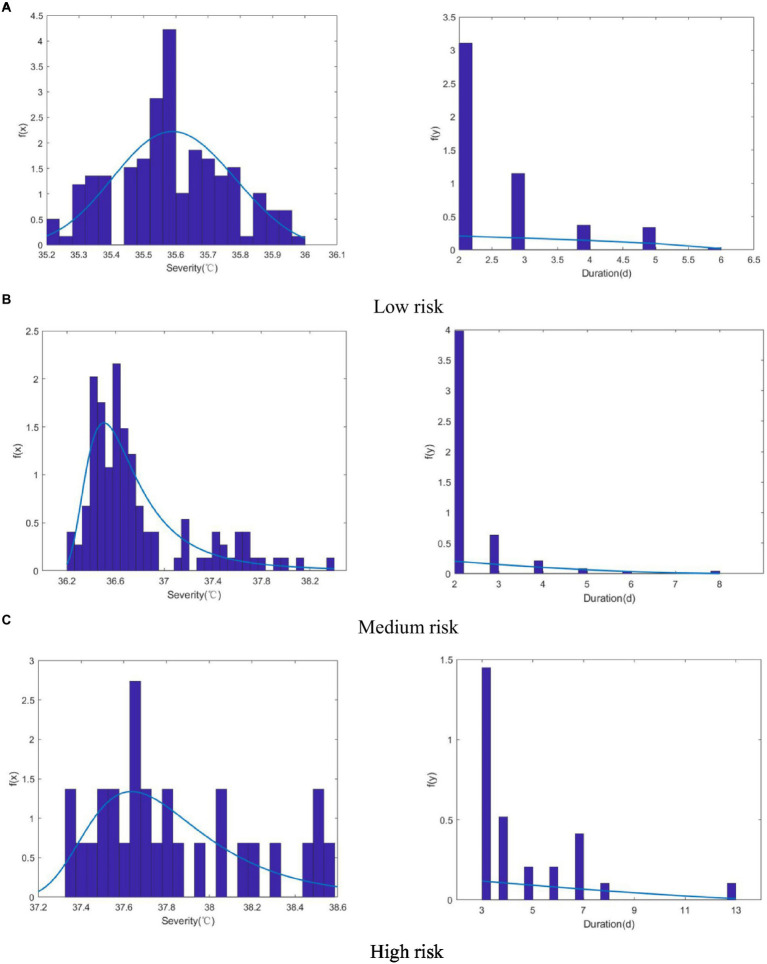
Comparison of the actual histograms and optimal probability density distributions of the 2 characteristics of heatwaves at 3 risk levels. **(A)** Low Risk; **(B)** Medium Risk;** (C)** High Risk.

Figure 5 compares the actual probability density distributions of the 2 characteristics of heatwaves with the optimal probability density distributions and shows that the distributions selected in this paper fit well with the actual characteristics of heatwaves.

The *t* copula of the elliptic copulas, the gaussian copula, and the Clayton copula, Gumbel copula, and Frank copula of the Archimedean copulas were selected to construct joint probability distribution functions of heatwave severity and duration for the different risk categories during the historical observation period. The parameters in the joint probability distribution functions of heatwave severity and duration were estimated using the maximum likelihood method. The goodness-of-fit tests were performed based on the maximum likelihood, AIC, BIC, and RMSE criteria, and the parameters and goodness-of-fit test results are shown in Table 5.

**Table 5:** Evaluation of copula parameters and goodness-of-fit tests for the different risk levels.

Risk level	Copula	Parameters	RMSE
Low risk	Gaussian	$\rho =\left[\begin{array}{cc} 1 & 0.12\\ 0.12 & 1 \end{array}\right]$	0.31
	*t*	$\alpha =\left[\begin{array}{cc} 1 & 0.28\\ 0.28 & 1 \end{array}\right],\mathrm{nu}=2442431$	0.26
	Clayton	***α*** = 0.42	0.25
	Frank	***α*** = 1.51	0.25
	Gumbel	***α*** = 1.11	0.39
Medium risk	Gaussian	$\rho =\left[\begin{array}{cc} 1 & -0.14\\ -0.14 & 1 \end{array}\right]$	0.17
	*t*	$\alpha =\left[\begin{array}{cc} 1 & -0.41\\ -0.41 & 1 \end{array}\right],nu=4669185$	0.25
	Clayton	***α*** = 0.26	0.28
	Frank	***α*** = −2.45	0.26
	Gumbel	***α*** = 1	0.2
High risk	Gaussian	$\rho =\left[\begin{array}{cc} 1 & 0.45\\ 0.45 & 1 \end{array}\right]$	0.13
	*t*	$\alpha =\left[\begin{array}{cc} 1 & 0.58\\ 0.58 & 1 \end{array}\right],nu=3.3649$	0.12
	Clayton	***α*** = 1.35	0.19
	Frank	***α*** = 3.74	0.13
	Gumbel	***α*** = 1.58	0.11

* ***ρ*** and *α***** are the linear correlation parameters in the copula, and nu is the degree of freedom.

According to the criterion that a small RMSE indicates a good copula fit, the Clayton copula exhibited the best fit for a binary copula of heatwave severity and duration in the low-risk category. The RMSE of the gaussian copula was significantly smaller than the rest in the medium-risk category, where it was the optimal joint probability distribution of heatwave severity and duration. For the joint distribution of heatwave severity and duration in the high-risk category, the goodness-of-fit of the Gumbel copula was the best.

**Figure 6:**
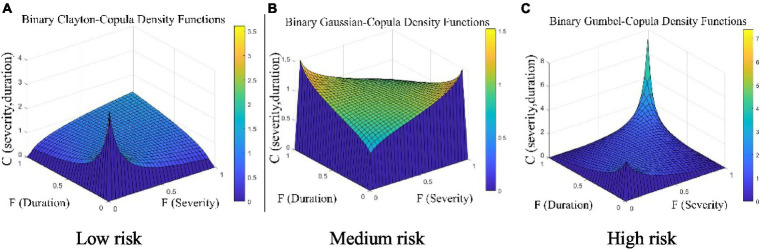
Joint probability density function plots of heatwave characteristics for the 3 risk levels **(A)** Low Risk; **(B)** Medium Risk; **(C)** High Risk.

Therefore, the Clayton copula, gaussian copula, and Gumbel copula functions were chosen to describe the 2-dimensional joint distribution of the severity and duration of low-, medium-, and high-risk heatwaves, respectively. The joint probability density functions of the heatwave characteristics in the 3 risk categories are shown in Figure 6.

Based on the data of heatwave severity and duration at different risk levels in the historical observation period, the co-occurrence return periods of heatwave severity and duration were calculated according to Eq.

**Figure 7:**
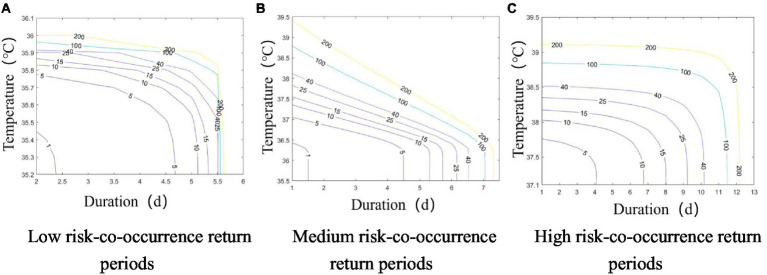
Co-occurrence return period plots for heatwave severity and duration at the 3 risk levels. **(A)** Low Risk; **(B)** Medium Risk; **(C)** High Risk.

The vertical axis is heatwave severity. The co-occurrence return period curves constructed from the heatwave severities and durations were used to compare heatwave severities from different risk categories with the same durations and co-occurrence return periods. For instance, a heatwave event with a duration of 3 days or more and a 5-year co-occurrence return period corresponded to temperatures of 35.74°C, 36.69°C, and 37.64°C, respectively, in the low-, medium-, and high-risk categories (see Figure 7).

When the duration of a low-risk heatwave event was 2d, the maximum heatwave severity was 36°C, and the corresponding co-occurrence return period was 149 years. In other words, the co-occurrence return period for heatwave events with severity greater than or equal to 36°C and a duration of 2d or more was 149 years in the low-risk category. When the duration of a medium-risk heatwave event was 2d, the corresponding maximum heatwave severity was 38.4°C, and its co-occurrence return period was 103.98 years. The maximum severity corresponding to a high-risk heatwave lasting 3d was 38.586°C, and the corresponding co-occurrence return period of the 2 characteristics was 50.6 years.

On the whole, as the heatwave severity and duration increased in each risk category, the co-occurrence return period gradually decreased, which meant that the probability of heatwave events increased.

### 4.3 Projection of future heatwave risks in Wuhan based on copulas

**Figure 8:**
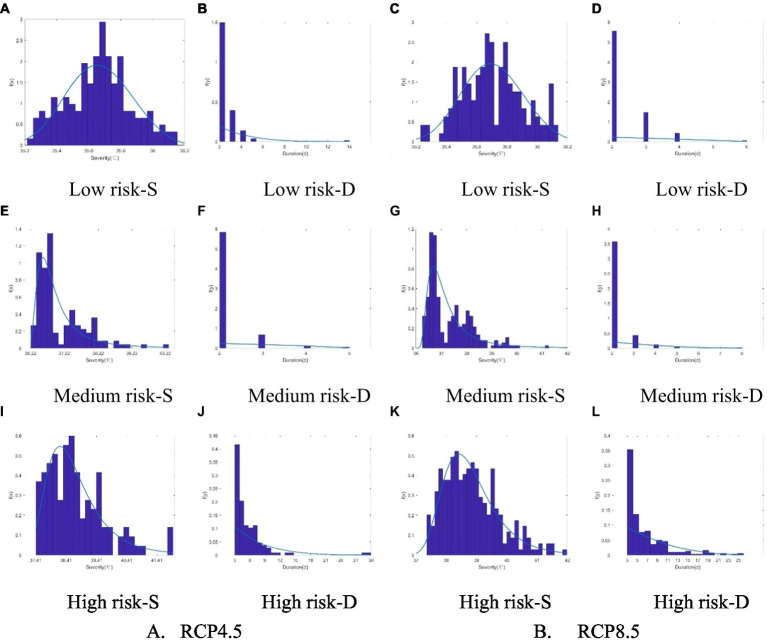
Comparison of the actual histograms and optimal probability density distributions of the 2 heatwave characteristics (S was severity, D was duration) under 2 emission scenarios and 3 risk levels. **(A-D)** Low Risk; **(E-H)** Medium Risk; **(I-L)** High Risk.

Table 6 shows the optimal marginal distributions for heatwave characteristics, i.e., severity (S) and duration (D), for the future period under the RCP4.5 and RCP8.5 emission scenarios. As in the historical observation period, the optimal marginal distributions fitted to the heatwave severity and duration in the low-, medium-, and high-risk categories were the GEV distribution and the GP distribution, respectively, and the parameters were again estimated using the maximum likelihood estimation method. Figure 8 compares the actual histograms of the 2 heatwave characteristics for the 2 emission scenarios and 3 risk levels with the optimal probability density distributions chosen. The performance was satisfactory, as in the historical observation period.

**Table 6:** Marginal distributions of heatwave characteristics under different scenarios and risk levels.

Emission scenario	Risk level	Characteristic	Marginal distribution	Parameters
RCP4.5	Low risk	S	GEV	[*k*,*σ*,*μ*] = [−0.27,0.2,35.59]
		D	GP	[*k*,*σ*,*μ*] = [−0.16,2.75,0]
	Medium risk	S	GEV	[*k*,*σ*,*μ*] = [0.41,0.37,36.69]
		D	GP	[*k*,*σ*,*μ*] = [−0.59,2.98,0]
	High risk	S	GEV	[*k*,*σ*,*μ*] = [0.1,0.68,38.27]
		D	GP	[*k*,*σ*,*μ*] = [−0.11,5.96,0]
RCP8.5	Low risk	S	GEV	[*k*,*σ*,*μ*] = [−0.26,0.19,35.63]
		D	GP	[*k*,*σ*,*μ*] = [−0.51,3.14,0]
	Medium risk	S	GEV	[*k*,*σ*,*μ*] = [0.39,0.48,36.82]
		D	GP	[*k*,*σ*,*μ*] = [−0.32,2.66,0]
	High risk	S	GEV	[*k*,*σ*,*μ*] = [0.01,0.72,38.41]
		D	GP	[*k*,*σ*,*μ*] = [−0.26,8.07,0]

*k: shape parameter, *σ*: scale parameter, *μ*: location parameter.

The *t* copula, gaussian copula, Clayton copula, Gumbel copula, and Frank copula were used to construct 2-dimensional joint distributions of heatwave severity and duration for the different risk levels under the RCP4.5 and RCP8.5 emission scenarios, and the parameter estimations were performed using the maximum likelihood estimation method. As in the historical observation period, the joint distributions were tested for goodness-of-fit using the max-likelihood, AIC, BIC, and RMSE criteria. The parameters and results of the goodness-of-fit tests are shown in Table 7.

**Table 7:** Evaluation of copula parameters and goodness-of-fit tests under different emission scenarios and risk levels.

	Risk level	Copula	Parameters*	RMSE
RCP4.5	Low risk	Gaussian	$\rho =\left[\begin{array}{cc} 1 & -0.06\\ -0.06 & 1 \end{array}\right]$	0.14
		t	$\alpha =\left[\begin{array}{cc} 1 & -0.35\\ -0.35 & 1 \end{array}\right],nu=2285091$	0.12
		Clayton	***α*** = 0.17	0.13
		Frank	***α*** = −2.06	0.12
		Gumbel	***α*** = 1	0.12
	Medium risk	Gaussian	$\rho =\left[\begin{array}{cc} 1 & -0.19\\ -0.19 & 1 \end{array}\right]$	0.16
		t	$\alpha =\left[\begin{array}{cc} 1 & -0.48\\ -0.48 & 1 \end{array}\right],nu=4671397$	0.23
		Clayton	***α*** = 0.39	0.26
		Frank	***α*** = −2.83	0.24
		Gumbel	***α*** = 1	0.25
	High risk	Gaussian	$\rho =\left[\begin{array}{cc} 1 & 0.58\\ 0.58 & 1 \end{array}\right]$	0.15
		t	$\alpha =\left[\begin{array}{cc} 1 & 0.66\\ 0.66 & 1 \end{array}\right],nu=3690875$	0.13
		Clayton	***α*** = 1.58	0.35
		Frank	***α*** = 4.61	0.09
		Gumbel	***α*** = 1.62	0.19
RCP8.5	Low risk	Gaussian	$\rho =\left[\begin{array}{cc} 1 & -0.07\\ -0.07 & 1 \end{array}\right]$	0.16
		t	$\alpha =\left[\begin{array}{cc} 1 & -0.26\\ -0.26 & 1 \end{array}\right],nu=12.2447$	0.16
		Clayton	***α*** = 0.17	0.18
		Frank	***α*** = −1.39	0.17
		Gumbel	***α*** = 1	0.17
	Medium risk	Gaussian	$\rho =\left[\begin{array}{cc} 1 & -0.26\\ -0.26 & 1 \end{array}\right]$	0.3
		t	$\alpha =\left[\begin{array}{cc} 1 & -0.53\\ -0.53 & 1 \end{array}\right],nu=3744169$	0.51
		Clayton	***α*** = 0.32	0.5
		Frank	***α*** = −3.44	0.54
		Gumbel	***α*** = 1	0.56
	High risk	Gaussian	$\rho =\left[\begin{array}{cc} 1 & 0.5\\ 0.5 & 1 \end{array}\right]$	0.14
		t	$\alpha =\left[\begin{array}{cc} 1 & 0.57\\ 0.57 & 1 \end{array}\right],nu=4669185$	0.15
		Clayton	***α*** = 1.05	0.34
		Frank	***α*** = 3.46	0.15
		Gumbel	***α*** = 1.47	0.13

* ***ρ*** and ***α*** are the linear correlation parameters in the copula function, and nu is the degree of freedom.

**Figure 9:**
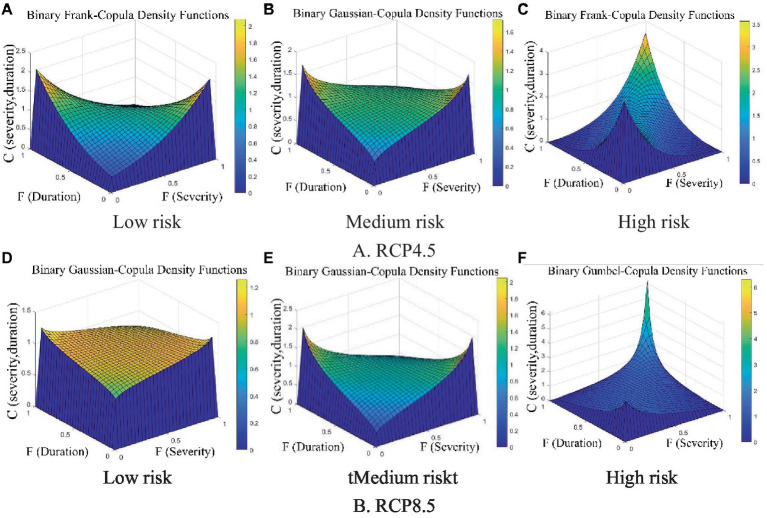
The joint probability density functions of heatwave characteristics under different scenarios and risk levels. **(A-C)** RCP4.5, **(D-F)** RCP8.5.

Based on the criteria used to select the best 2-dimensional copula functions in the historical observation period, for the low- and high-risk categories under the RCP4.5 emission scenario, the Frank copula fit best; for the medium risk under the RCP4.5 emission scenario, the gaussian copula was best fit. The gaussian copula also had the best fit for the low- and medium-risk categories under the RCP8.5 emission scenario, and the Gumbel copula fit best for the high risk category. The joint density functions of heatwave characteristics under the different scenarios and risk levels are shown in Figure 9.

**Figure 10:**
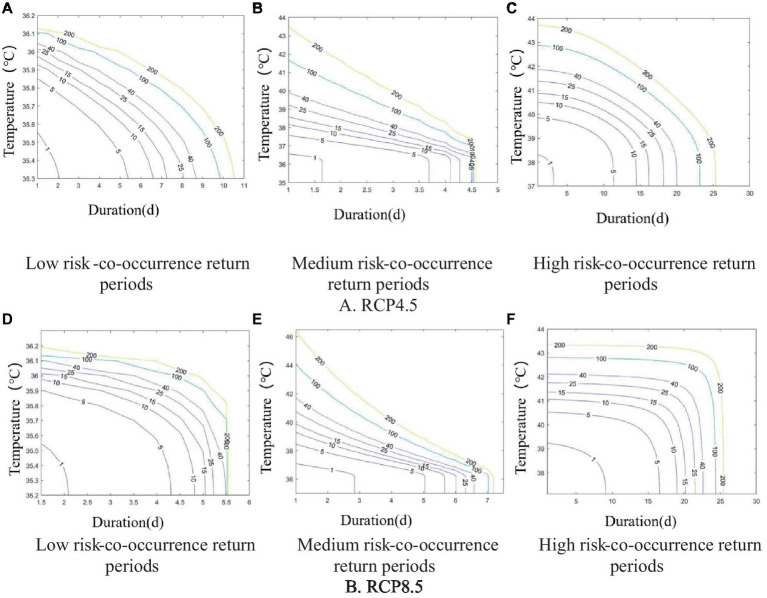
Co-occurrence return period plots of heatwave intensity and duration for 3 risk levels and 2 scenarios. **(A-C)** RCP4.5; **(D-F)** RCP8.5.

The return periods of heatwaves with the 3 different risk levels under the RCP4.5 and RCP8.5 emission scenarios are shown in Figure 10. The characteristics of the co-occurrence return period curves are the same as during the historical observation period. The co-occurrence return period for each risk level in both emission scenarios tended to increase as heatwave severity intensified and heatwave duration lengthened, which matches the frequency characteristics of heatwaves in the historical observation period.

In the RCP4.5 emission scenario, the maximum heatwave severity in the low-risk category was 36.155°C when the heatwave lasted for 2d, corresponding to a co-occurrence return period of 359.27 years. The maximum heatwave severity was 40.315°C when the heatwave lasted for 2d in the medium-risk category, which corresponded to a co-occurrence return period of 101.39 years for this type of event. The maximum heatwave severity for a high-risk heatwave event with a duration of 7 days was 41.93°C, corresponding to a co-occurrence return period of 57.74 years.

In the RCP8.5 emission scenario, the maximum heatwave severity (36.14°C) and duration (2d) in the low-risk category, maximum heatwave severity (41.26°C) and duration (2d) in the medium-risk category, and maximum heatwave severity (41.97°C) and duration (19d) in the high-risk category corresponded to co-occurrence return periods of 89.25, 68.5, and 51.88 years, respectively.

Compared with the co-occurrence return periods of the 3 heatwave risk levels in the historical observation period, the co-occurrence return periods of each heatwave risk gradually shortened as the concentrations of greenhouse gas emissions increased in the RCP4.5 and RCP8.5 emission scenarios. This implies that longer and more intense heatwaves will occur more frequently as greenhouse gas levels continue to increase. For instance, the co-occurrence return period corresponding to the most severe intensity and duration of a low-risk heatwave in the observation period was 149 years, whereas the return periods for a heatwave of the same intensity and duration in the RCP4.5 and RCP8.5 scenarios were 34.81 years and 14.87 years, respectively. For a medium-risk heatwave event, the co-occurrence return period corresponding to the most severe intensity and duration in the observation period was 103.98 years, whereas in the RCP4.5 and RCP8.5 scenarios, it was 23.95 years and 9.10 years, respectively. Likewise, the co-occurrence return period corresponding to the maximum heatwave intensity and duration for a high-risk event in the observation period was 50.6 years, and it was 1.34 years and 0.56 years in the RCP4.5 and RCP8.5 scenarios, respectively. All 3 scenarios show that increased greenhouse gas emissions lead to more frequent heatwaves.

## 5 Conclusion and discussion

Global climate change is leading to increasing heat waves in major cities, with serious impacts on human health and life on Earth (39). Traditional meteorological warnings are based on the strength of the heat signal and do not consider the possible physical health risks associated with heat exposure. A heat-health warning system has been shown to effectively reduce premature deaths caused by heatwaves and is only in use in a few high-income countries (40). There is an urgent need to consider the potential health risks due to heat exposure in the heatwave definition, classification, and warning systems for the health protection of vulnerable populations in most low-income and middle-income countries. Therefore, in this study, nine heat wave definitions that can well capture the effects on non-accidental mortality of heatwaves in Wuhan were introduced and classified into low, medium, and high risk levels to investigate the historical and future likely changes of heatwave risks. Copula functions were used to analyze the joint probability distributions of heatwave severity and duration and to project the future heatwave co-occurrence interval.

Daily maximum near-surface air temperature projections based on 4 CMIP5 climate models under RCP4.5 and RCP8.5 scenarios were collected for future heatwave risk assessment. Due to the complexity of the climate system and the differences in the physical processes integrated with the model, the simulation results may differ from actual observations and between models. Therefore, it is necessary to conduct a comprehensive and quantitative evaluation of the model products before making predictions about future climate. The Taylor diagrams and the S-skill score were used to evaluate the performances of four GCMs in simulating temperature variables in Wuhan. The results showed that CMIP5 models had satisfying performance in reproducing observed characteristics of temperature extremes in Wuhan, and the IPSL-CM5A-LR and MIROC5 were the best-fitted models under the RCP4.5 and RCP8.5 emission scenarios, respectively, with a high S-skill score of 0.99.

The identification of heatwave events in the 3 risk categories during the observation and future periods found that (a) the frequency of heatwave events increased along with increasing concentrations of greenhouse gas emissions; (b) the frequency of heatwave events in the high-risk category increased significantly; (c) the frequency of heatwave events in the low-risk category increased but not significantly. Overall, the co-occurrence period gradually got longer with increasing heatwave intensity and heatwave duration at all risk levels. Compared to RCP4.5, the return period for each risk category of heatwave became progressively shorter under the RCP8.5 scenario, leading to more frequent heatwaves. This paper recommends that policymakers prioritize responses to extreme heat events and implement public health measures to reduce the health risks associated with local heatwaves. In addition, this study provides warnings for cities with climates similar to that of Wuhan, China, and provides useful references for facing heat disaster risks.

## Data availability statement

The original contributions presented in the study are included in the article/supplementary material, further inquiries can be directed to the corresponding authors.

## Author contributions

SC: Conceptualization, Funding acquisition, Methodology, Writing – original draft. JZ: Software, Validation, Writing – original draft. HD: Visualization, Writing – original draft. ZY: Data curation, Writing – original draft. FL: Resources, Supervision, Writing – review & editing. JB: Data curation, Funding acquisition, Writing – review & editing. SK: Conceptualization, Funding acquisition, Writing – review & editing.

## Footnotes

^1^ http://data.cma.cn/

^2^ https://esgf-node.llnl.gov/search/cmip5/
